# Fusing compressed deep ConvNets with a self-normalizing residual block and alpha dropout for a cost-efficient classification and diagnosis of gastrointestinal tract diseases

**DOI:** 10.1016/j.mex.2022.101925

**Published:** 2022-11-14

**Authors:** Francis Jesmar P. Montalbo

**Affiliations:** College of Informatics and Computing Sciences, Batangas State University, Batangas, Philippines

**Keywords:** Alpha dropout, Biomedical Imaging, Deep convolutional neural networks, Endoscopy, Feature fusion, Image classification, Residual learning, Self-normalization, SeLU

## Abstract

The challenging task of diagnosing gastrointestinal (GI) tracts recently became a popular research topic, where most researchers performed extraordinary feats using numerous deep learning (DL) and computer vision techniques to achieve state-of-the-art (SOTA) diagnostic performance based on accuracy. However, most proposed methods relied on combining complex computational methods and algorithms, causing a significant increase in production difficulty, parameter size, and even training cost. Therefore, this method proposes a straightforward approach to developing a vision-based DL model without requiring heavy computing resources or reliance on other complex feature processing and learning algorithms. This paper included the step-by-step procedure consisting of network compression, layer-wise fusion, and the addition of a modified residual layer (MResBlock) with a self-normalizing attribute and a more robust regularization. In addition, the paper also presents the performance of the proposed method toward the diagnosis of four GI tract conditions, including polyps, ulcers, esophagitis, and healthy mucosa. The paper concludes that the proposed method did radiate a significant improvement in the overall performance, cost-efficiency, and especially practicality compared to most current SOTA methods.

• The proposed method combined profound techniques like feature fusion, residual learning, and self-normalization to develop a lightweight model that accurately diagnoses gastrointestinal (GI) tract conditions.
• The model produced from the proposed method generated better performance than most pre-existing state-of-the-art Deep Convolutional Neural Networks that diagnosed the presented four GI tract conditions.
• Aside from its competitive performance, the model based on the proposed method only had 1.2M parameters and only consumed 1.5 GFLOPS, making it significantly more cost-efficient than most existing solutions.

## Graphical abstract

**Figure:**
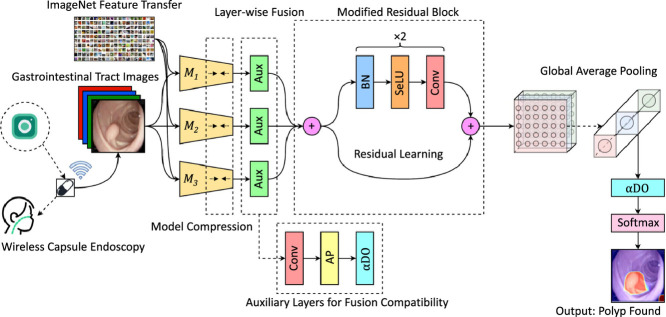
Automated Detector and Classifier of Gastrointestinal Anomalies or Diseases from Endoscopic Images using a Deep Learning-based Model.

Specifications table

**Table:** 

Subject Area:	Computer Science
More specific subject area:	*Deep Convolutional Neural Networks and Medical Image Diagnosis*
Method name:	A Novel approach to reducing the cost of training for a feature fused Deep Convolutional Neural Network using network compression, residual learning, and self-normalization techniques.
Name and reference of original method:	The proposed method consists of numerous techniques discussed and cited within the method details of this article.
Resource availability:	All resources in the link below include the codes for building, training, validating, and testing the model and the dataset. https://github.com/francismontalbo/mfurecnn

## Method details

With the growing recognition of Deep Learning (DL) and Computer Vision (CV), several studies began to incorporate them in solving complex medical imaging endeavors [1]. Recently, researchers had the notion of combining both methods to automate the diagnosis of gastrointestinal (GI) diseases from images captured through Wireless Capsule Endoscopy (WCE) [2]. Upon evaluation, they discovered that these technologies, based on their diagnostic accuracy, can equate to an expert that has the potential to advance medical imaging to a whole new level. According to various studies, DL and CV attained such feats because of Convolutional Neural Networks (CNN) [3].

CNN is a CV model comprised of interlinked layers that utilize different numerical procedures to extract and understand patterns from a particular image dataset. Simultaneously acting as a DL model, CNNs perform feature extraction through a feed-forward fashion and stochastically compound and adjust weights on those extracted features using a loss function during backpropagation [4]. In the later years after the release of CNN, though it had shown substantial improvements in image classification, most researchers noticed its weakness in dealing with large-scale datasets with hundreds to thousands of classes and instances. Based on successive research, others commenced proposing the addition of more layers to boost its performance, in which AlexNet successfully surpassed the performance of a typical CNN. According to their assessment, the added layers significantly contributed to the enhanced results in AlexNet [5]. Following researchers then utilized such a method leading to a deeper and more accurate model like the VGGNet [6]. Due to the popularity and potency of layer deepening, researchers began to progress them even more and eventually referred to them as Deep CNNs (DCNN) [7]. However, such a concept of deepening the architecture of a DCNN model eventually resulted in a saturated performance, indicating a possible roadblock. Therefore, researchers again continued their pursuit of developing a better solution that could solve such a problem.

Lately, DCNNs has become one of the hottest topics in research and medical imaging in such a short period, spawning newer approaches with state-of-the-art (SOTA) calculations that redefined their generation of features. With numerous proposals, K. He et al. formulated a revolutionary theory called residual learning that solved the saturation problem. According to their solution, they constructed a model known as ResNet that incorporates their theory of using skip connections, making ResNet learn only from residuals. Based on their empirical results and findings, their proposed ResNet model, compared to a non-residual model, achieved better performance even with more layers, showing a significant result that can alleviate the saturation problem found in non-residual DCNN models [8]. With the success of residual learning, most DCNNs currently incorporate it within their architectures, helping them reach SOTA performance in multiple tasks even with thousands of classes and millions of images [7].

Though DCNNs proved their capacity to perform complex classifications, they still rely heavily on data to generate sufficient learnable features, even though they already consist of numerous extraction layers. In some cases, DL models trained with inadequate data tend to become less accurate than traditional machine learning (ML) models trained on handcrafted features [9]. Fortunately, a recent study introduced the importance of having self-normalizing properties in DL models to generate robust abstract representations. To make their theory possible, they proposed the addition of the Scaled exponential Linear Units (SeLU), an activation function based on the Banach fixed-point theorem that reduces or eliminates the occurrence of the vanishing and exploding gradients problem in most deep-layered models. To support their claims, they trained a DL model with self-normalizing properties acquired from SeLU, which they discovered could train more effectively even with more layers than those with lesser layers and without SeLU. In addition, based on their evaluated results, they found that SeLU could perform better than the conventional Rectified Linear Unit (ReLU) as it contributes better convergence when paired with an alpha dropout (DO) [10].

As identified, most proposed solutions still require decent or high-end computing resources to reproduce, making them less viable for real-world applications. Unfortunately, even expensive DCNN models with numerous layers can still perform closely like or less than conventional ML algorithms trained with handcrafted features. Therefore, this paper proposes a method to retain cost-efficiency in DL while producing a robust set of features from a limited dataset. The proposed method incorporates a variety of approaches, including model selection, network compression, layer-wise fusion, and residual learning imbued with self-normalizing properties. These approaches aim to reduce the cost of production and deployment, generate a broader spectrum of features from limited data even with a smaller network, and deliver better regularization to minimize overfitting.

## Model selection

Before heading straight to a layer-wise fusion, the proposed method focuses first on what models to fuse. It is worth mentioning that this paper does not cover how to select the best model combination to fuse, as data itself can shift, and DL models may react differently towards a specific task due to its stochastic and sometimes indefinite nature [11]. Nevertheless, this paper chose models that explicitly considered performance and cost-efficiency. The following models include the EfficientNet, MobileNet, and ResNet. The intuition for having different models instead of the same lies in the importance of feature diversity and the prevention of feature redundancy.

ResNet [8,12] became one of the selected models for its capability of alleviating the diminishing and exploding gradient problem in deep-layered networks. The concept of ResNet utilizes the use of a skip-block that produces an identity map referred to as a residual, allowing deep-layered models to retain or even improve performance during training.

Even with the success of DCNN models in performing tasks in CV, most of them still demand expensive computing requirements. Hence, MobileNet [13] became a vital component in this method due to its novel approach to reducing the computation cost using pointwise and depthwise convolutions (DWConv).

With the growing demands for ubiquitous DL, EfficientNet [14] developed a DCNN model that can flexibly adapt to datasets with different image dimensions and resolutions for improved performance. The EfficientNet also relies on an inverted residual block originating from MobileNetV2 and ResNets. With such characteristics, EfficientNet became of the selected models for this paper.

To better elaborate the specifications and recognition of each selected model, Table 1 presents their parameter size, ImageNet benchmark top-1 and top-5 accuracies, parameter size, disk space requirement, and inference performance in time via GPU. Based on the specifications given, the EfficientNetB0 shows that it performed better than the other two models with the ImageNet data that consist of 1,000 images with millions of instances. On the other hand, though not as accurate, MobileNetV2 shows how fast and lightweight it is compared to the two. Though the ResNet50V2 falls behind the two models based on the given metrics, we should know that ResNet50V2 and its residual learning concept helped them attain such scores, showing how it generally elevated DL and CV. ResNet50V2 also exemplifies that it still had fewer parameters than a VGGNet with >130M parameters [6].

**Table 1:** Specifications of the selected models for fusion.

Model	Disk Size	Top-1 Accuracy	Top-5 Accuracy	Inference speed	Params
EfficientNetB0	29MB	77.1%	93.3%	4.9ms	5.3M
ResNet50V2	98MB	76.0%	93.0%	4.4ms	25.6M
MobileNetV2	14MB	71.3%	90.1%	3.8ms	3.5M

## Network compression

After identifying the models for diagnosing GI tracts, each underwent compression to have its parameters reduced further. Due to their complex architectures, defining a specific layer to remove can become appalling. Therefore, each model received its truncation based on its core blocks rather than distinguishing a specific cut-point layer-by-layer. Based on recent studies, model compression or truncation has proven that DCNN models trained in a significantly smaller dataset than the ImageNet can still perform adequately with reduced overfitting. Such a finding led to the development of low-cost DCNNs that can fit easily in most devices [15], [16], [17], [18].

In Table 2, the following presents the specifications of each selected model, including their compressed parameters and features after the compression, the status of their layers during training, their respective cut-point layer, and the number of blocks deducted from their base architecture. Though these cut-points do not precisely pertain to their optimal cut-points, the selected cut-points still yielded a significant performance while focusing on the number of features to retain and the number of parameters to reduce. It is worth mentioning that these cut-points can be changed. Hence, this method does not restrict only to the given, as cut-points can increase to a deeper or shallower cut depending on the available resources.

**Table 2:** Compression settings.

Model	Initial	Compressed	Features	Status	Cut-point	Deducted
EfficientNetB0	4M	2.9M	192	Frozen	‘block6d_add’	1
MobileNetV2	2.2M	558K	96	Frozen	‘block12a_add’	4
ResNet50V2	23M	1.1M	512	Frozen	‘conv3_block3_out’	2

**Fig. 1:**
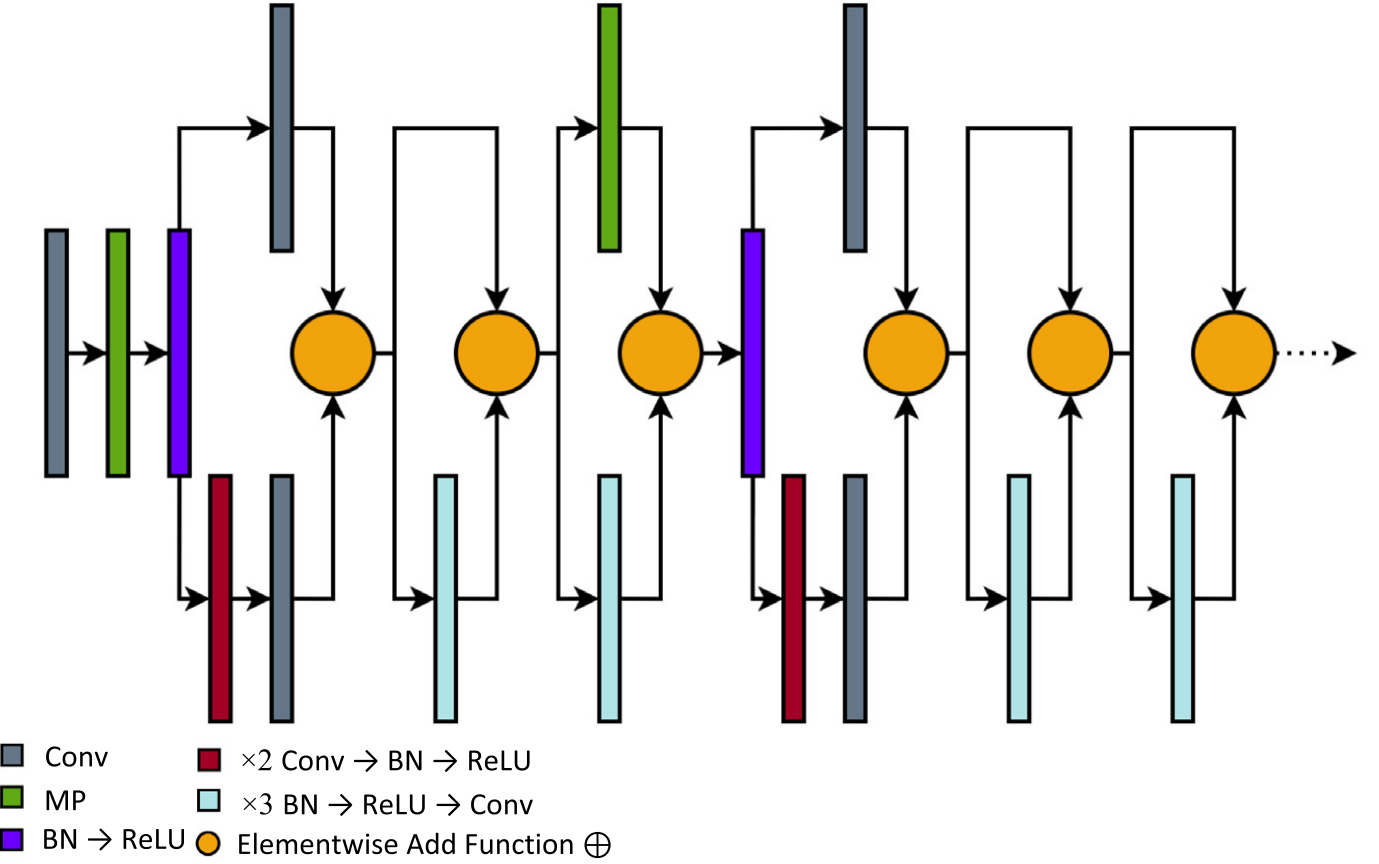
The architecture of the compressed ResNetV2 model.

Fig. 1 illustrates the compressed ResNetV2 model that shows an observable pattern based on its base model, the ResNet50V2. However, unlike the original, the compressed version had lesser core blocks. Therefore, making its parameter size significantly smaller. The primary components of the compressed ResNetV2 consist of an entry block composed of a Convolution (Conv) → Max-Pool (MP) → Batch Normalization (BN) → ReLU layers connected to a residual branch of × 2 Conv → BN → ReLU layers connected to a Conv layer ⊕ with another Conv layer. The succeeding component includes a branch of × 3 BN → ReLU → Conv ⊕ with the previous outputs. Then another residual branch with a × 3 BN → ReLU → Conv follows, but instead, ⊕ with an MP layer connected to the previous output [8,12]. Lastly, the final layers before the cut-point consist of two successive × 3 BN → ReLU → Conv like the second block.

For better reproducibility and in-depth details, this link provides the exact specifications and design of the compressed ResNetV2: https://github.com/francismontalbo/mfurecnn/blob/main/graphics/compressed_resnet.png.

**Fig. 2:**
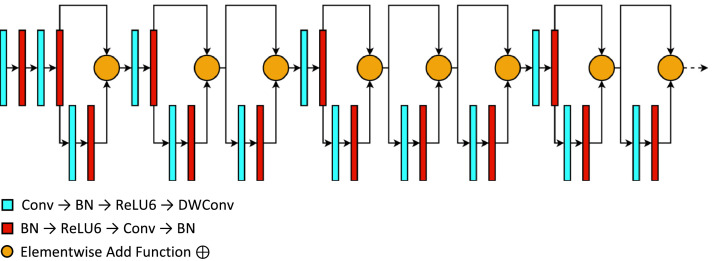
The architecture of the compressed MobileNetV2 model.

As illustrated in Fig. 2, the compressed MobileNetV2 presents a repeating set of layers designed in a specific pattern that originated from the base model. The compressed architecture with an MBConv layer allows rapid, straightforward, and lightweight computations that produce robust features, making MobileNetV2 more accessible for most mobile devices. The MobileNetV2 also incorporates a skip connection and a ⊕ function with its core layers composed of a Conv → BN → ReLU6 → DWConv and a BN → ReLU6 → Conv → BN. Unlike the regular ReLU function, the modified ReLU6 limits the activation of features to only an arbitrarily selected value of 6. Such modification added further compression and reduced the complexity of a typical ReLU [13].

This link provides the complete specification and overview of the compressed MobileNetV2: https://github.com/francismontalbo/mfurecnn/blob/main/graphics/compressed_mobilenet.png.

**Fig. 3:**
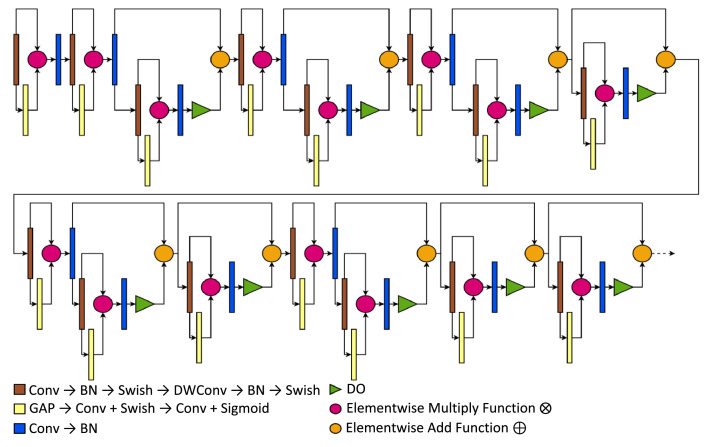
The architecture of the compressed EfficientNet model.

In Fig. 3, the following presents the core structure of the EfficientNetB0 model, which primarily consists of the MBConv layer. The MBConv layer presents an inverted bottleneck residual originating from the MobileNetV2′s concept with added squeeze-and-excitation blocks that use lightweight computations. These combinations conveyed better proficiency for feature representation in EfficientNets. As illustrated in the EfficientNet's version, the squeeze-and-excitation block consists of a Global Average Pooling (GAP) followed by a Conv layer activated by Swish, a multiplication of a linear and the sigmoid activation function that had better performance than the conventional ReLU [19]. The following Conv layer then connects to another Conv activated by a Sigmoid, setting a smooth gating function. After the said Conv layer, the squeeze-and-excitation connects to a DO layer for added regularization ⊕ with the previous Conv → BN layers. The combinations of these approaches made EfficientNet one of the most cost-efficient DCNN and highly accurate models in recent years [20].

This link presents a more detailed overview of the compressed EfficientNet's architecture: https://github.com/francismontalbo/mfurecnn/blob/main/graphics/compressed_efficientnet.png.

## Layer-wise fusion with auxiliary layers

After the proposed layer reduction, it compressed the models to become less complex and significantly lighter than before. However, at this point, a layer-wise fusion is still not possible due to the unequal output shapes of the compressed models. Therefore, to alleviate such a problem, the proposed method also provided each model with its respective auxiliary layers tailor-fitted to reshape their incompatible cut-points, making a layer-wise fusion possible.

Table 3 presents the specifications of the mentioned auxiliary layers of each model composed of a Conv and Average Pooling (AP) layer. In this paper, the selected output shape became 7^2^ × 192, as the filter size *f* of 192 provides better adequacy than 96, but still less than 512, as the proposed method considers the importance of cost. By providing the specific kernel size *k*, strides *s*, pool size *p,* and other settings after the cut-points of each model, they eventually had a similar output shape of 7^2^ × 192, making them compatible for a layer-wise fusion [21]. On the other hand, the auxiliary layers also contain an alpha DO with a rate of 0.2 that improves the regularization of the incoming fused features but does not affect the reshaping of each model's output. If cases that other researchers or users use cut-points that do not reflect the ones in this proposed method, the Conv and AP settings will necessitate alterations to make them fit [21].

**Table 3:** Specifications of the proposed auxiliary layers for the layer-wise fusion.

Model	Shape	Conv Layer Specifications	AP	αDO
EfficientNetB0	7^2^ × 192	*f*=192; *k*=1; *s*=1; Padding=Valid; activation=SeLU; initializer=LeCun Norm	*p*=1; *s*=1; Padding=Valid	Rate=0.2
MobileNetV2	14^2^ × 96	*f*=192; *k*= 8; *s*=1; Padding=Valid; activation=SeLU; initializer=LeCun Norm	*p*=1; *s*=1; Padding=Valid	Rate=0.2
ResNet50V2	28^2^ × 512	*f*=192; *K*=6; *s*=1; Padding=Valid; activation=SeLU; initializer=LeCun Norm	*p*=3; *s*=3; Padding=Valid	Rate=0.2

## Modified residual block

Once the models had their final layers or cut points fused. This section presents the insertion of a residual skip block after the fusion layer to handle the fused features, decreasing the chance of possible overfitting to generate better performance. Unlike the original ResNetV2 block [12], this method proposes a modified residual block (MResBlock) that integrates self-normalization and regularization, which consists of × 2 BN → SeLU → Conv.

After the layer-wise fusion, each selected model had its *M_j_* outputs fused, which served as the *x* input for the MResBlock (1).

(1) $$x=\sum_{j=1}^{M}j$$

Subsequently, in (2), the MResBlock creates a residual map *y* using a residual function *F* that takes the fused inputs *x* in conjunction with its weights *W*. However, during the generation of *y*, the summed features must become equal throughout the MResBlock. Therefore, the MResBlock also included a square matrix *W_q_x*, making their output shapes equal and model fitting possible [8,12].

(2) $$y=F\left(x,\;\left\{W_{i}\right\}\right)+W_{q}x$$

Considering that the task pertains to multi-class classification, the proposed method generated the residual maps using the residual function *F* based on (3). In addition, to incorporate robust abstract representations within the residuals, this method substituted the original ReLU with a self-normalizing function called SeLU [10,22].

(3) $$F=W_{n}SeLU\left(W_{1}x\right)$$

## Self-normalizing activation (SeLU)

Based on the MResBlock, as a replacement for ReLU, this method uses a SeLU function, which emanates better cost-efficiency than ReLU while preventing gradients from dying or vanishing. Presented in (4), SeLU performs normalization by retaining the mean and variance values at 0 and 1, adding better regularization during training, where *x* signifies α = 1.6733 and λ = 1.0507 [10,22].

(4) $$SeLU\left(x\right)=\lambda \left\{ \begin{array}{c} x,\;if\;x>0\\ \alpha e^{x}-\alpha ,\;if\;x\leq 0 \end{array}\right.$$

## Alpha dropout

As defined, DO enhances a DL model's capacity to counteract overfitting problems that can cause performance reduction. The typical DO performs the random shutting of neurons at the fully connected layers by setting their values to zero with 1-*p*. According to some studies, DO works effectively with a ReLU activation as it drops values to zero, making them reach a low variance territory that is beneficial to ReLU. However, both do not possess self-normalization due to their low variance reaching a max value of zero, whereas SeLU retains a low non-zero variance (5). Therefore, making the standard DO less potent and impractical. As shown, sets that randomly input values to α′ imply the effectiveness of the alpha DO. These qualities show why alpha DO suits SeLU better, as it restores the initial mean and variance values while preserving self-normalization during training, whereas the standard version does not [10,22].

(5) $$lim_{x\to \infty }SeLU\left(x\right)=-\lambda \alpha =\alpha^{\prime }$$

## Model construction and training settings

**Fig. 4:**
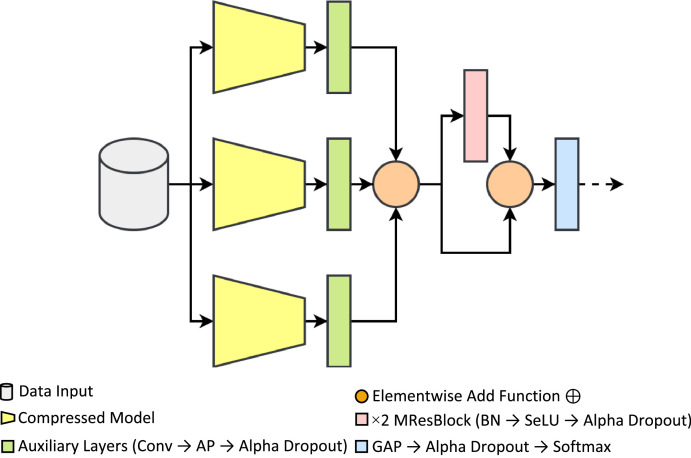
Model architecture based on the proposed method.

With the proposed method, it managed to build a compact architecture that incorporated feature fusion, residual learning, self-normalization, and enhanced regularization properties. Unlike the other methods proposed, this work did not require training or running other models or pipelines, as it can train similarly to an ordinary end-to-end DCNN model, as shown in Fig. 4. It is worth noting that the following compressed models in the proposed method received pre-trained features from ImageNet via transfer learning.

Table 4 presents the selected hyper-parameter settings to train the proposed model. It is worth noting that the selected hyper-parameters did not come from any optimization algorithms. Instead, they are only empirically tuned based on the existing computing resources and commonly used values when training a DCNN model. Therefore, other researchers can still impose changes to the values if necessary. Further, this approach emphasizes that the proposed cost-efficient method would not require costly tuning methods to produce a well-performing model to diagnose GI tracts.

**Table 4:** Hyper-parameters of choice.

Hyper-Parameter	Value
Batch Size	8
Epochs	25
Optimizer	Adam
Learning Rate	0.00001

In a DL model, due to its stochastic learning approach, the Learning Rate (LR) hyper-parameter plays a vital role in how a model can quickly adapt to a given set of data [23]. However, once the model starts to train, the initial LR value can no longer change, requiring another set of LR for the subsequent training. This process can become time-consuming and expensive, as the model tends to learn inadequately due to either a very high or very low LR, making the process repetitive. Therefore, this method included a callback function called ReduceLROnPlateau that adjusts the LR every time the results do not improve after a series of epochs [24].

Further, using a *patience* parameter, the ReduceLROnPlateau callback gets notified when it needs to adjust the LR. In this method, the *patience* parameter had a value of two, indicating that the ReduceLROnPlateau needs to decrease the current LR by half if the accuracy did not improve after two epochs (6). However, if in case that the LR hits a value too low, the model might no longer learn throughout the entire training period. Therefore, the callback function also had an LR limit of 0.000001 to ensure that the LR does not plunge too low and cause resource wastage during training.

(6) $$LR_{new}=LR*0.5$$

During training, a model necessitates a loss function to tweak the weights it provides to the extracted features. Knowing that the problem follows a logistic multi-class problem, the suitable loss function became the Categorical Cross-Entropy Loss *CCE_loss_* (7) [25].

(7) $$CCE_{loss}=-\sum_{c=1}^{m}Y_{o,\;c}log\left(P_{o,\;c}\right)$$

## Method validation

The dataset selected to evaluate the proposed method includes the KVASIR [26] and ETIS-Larib Polyp DB [27] datasets. These datasets came from highly reputable sources collected using the WCE technology and labeled by medical experts in the given field.

Table 5 presents the following image samples included in the dataset, the total number of samples for each class in the train, validation, and test set. Before entering the model pipeline, these images' pixels were rescaled by 1.0 / 255, lessening cost and preventing the possibility of poor convergence. In the conducted experiments, the train samples received augmentations during training, whereas the validation and test did not. Executing the augmentation during training instead of directly adding augmented samples allows the training approach to become more flexible and accessible, where other users can train the model with or without augmentation. In addition, this approach prevents data leakage when intervening with the images during data preparation, which can raise the possibility of ruining the evaluation of the model.

**Table 5:** Specification of the curated gastrointestinal tract endoscopy images.

Raw samples	Class	Train	Validation	Test	Total
	Normal	800	500	200	1500
	Ulcer	800	500	200	1500
	Polyps	800	500	200	1500
	Esophagitis	800	500	200	1500
Total		3,200	2,000	800	6,000

For better cogency of the proposed method, this article included an ablation study highlighting the implication of feature fusion, model compression, and self-normalization with added regularization. The following presents the performance comparison and an overall assessment of the ideal model produced with the proposed method. In this article, the metrics used to measure a model's performance included accuracy (8), precision (9), recall (10), and F1-score (11), calculated based on the following equations below. As presented, each metric is calculated based on the instances of GI tracts diagnosed if they are True Positive (TP), True Negative (TN), False Positive (FP), and False Negative (FN) [28].

(8) $$accuracy=\frac{TP+TN}{TP+TN+FP+FN}$$

(9) $$precision=\frac{TP}{TP+FP}$$

(10) $$recall=\frac{TP}{TP+TN}$$

(11) $$f_{1}-score=\frac{2*precision*recall}{precision+recall}$$

The following presents an ablation study to exemplify the proposed method's importance in diagnosing four GI tract conditions. The ablation consisted of different versions of the fused model with and without the MResBlock and conditions of having an alpha DO, standard DO, and without a DO. This approach expounds on the effectiveness of residual learning, self-normalization, and regularization for a fused model. As presented in Table 6, the proposed method produced the best results from the model trained with an MResBlock, SeLU activation, and alpha DO. In contrast, models without those exact stipulations attained a lesser performance. Therefore, indicating the collective potential of residual learning, self-normalization, and an improved regularization in providing significant performance improvements to a fused GI tract diagnostic model.

**Table 6:** Ablation results from the validation dataset.

Accuracy	Precision	Recall	F1-Score	MResBlock	SeLU	Alpha DO	DO
96.65%	96.67%	96.65%	96.65%	✓	✓	✓	
95.00%	95.08%	95.00%	95.00%	✓	✓		✓
93.20%	93.66%	93.20%	93.19%	✓	✓		
94.55%	94.64%	94.55%	94.54%			✓	
93.35%	93.80%	93.35%	93.33%				✓
94.35%	94.56%	94.35%	94.32%				

Likewise, the model trained with the MResBlock, SeLU, and alpha DO remain the most accurate model even with different samples. As presented in Table 7, the said model attained 97.75% across the entire metrics, showing how extensively the proposed components affected its overall performance. Additionally, considering the other deviations with an MResBlock and SeLU without or with the standard DO, they also showed better performance than those without the MResBlock and SeLU.

**Table 7:** Ablation results from the test dataset.

Accuracy	Precision	Recall	F1-Score	MResBlock	SeLU	Alpha DO	DO
97.75%	97.75%	97.75%	97.75%	✓	✓	✓	
97.12%	97.15%	97.12%	97.12%	✓	✓		✓
94.13%	94.52%	94.13%	94.09%	✓	✓		
96.13%	96.17%	96.12%	96.12%			✓	
94.50%	94.87%	94.50%	94.47%				✓
95.13%	95.25%	95.12%	95.08%				

Based on the ablation study, the results highlight that the proposed MResBlock, SeLU, and alpha DO did draw additional improvements for the fused model. However, isolating only the performance comparison against its variants does not provide sufficient evidence considering its contribution to GI tract diagnosis. In addition, concentrating alone on performance would not emphasize its cost-efficiency and practicality. Therefore, this article compares the fused model trained with the proposed method versus other SOTA DCNNs using the equivalent datasets based on accuracy, parameter size, and FLOPs.

**Fig. 5:**
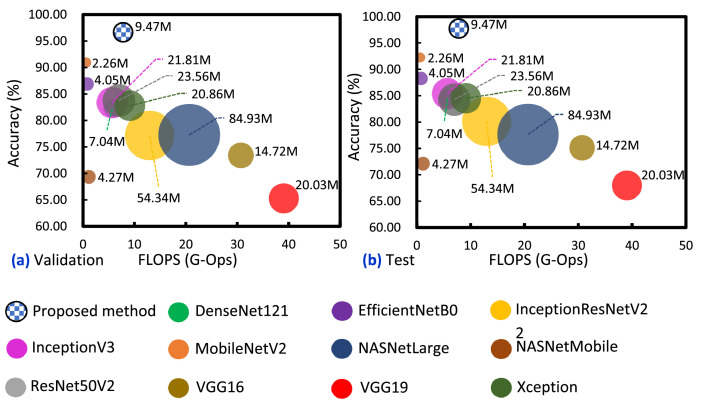
Cost-efficiency to accuracy ratios comparison.

As illustrated in Fig. 5, the proposed method yielded the most accurate model with the least number of parameters and FLOPs. Though it still had 9.47M parameters, which is higher than MobileNetV2 with 2.26M, EfficientNetB0 with 4.05M, and NASNetMobile [29] with 4.27M, comparing its performance against those models still makes the trade-off reasonable. To support such a claim, the smaller models only attained an overall accuracy of <92%, whereas the fused model trained with the proposed method achieved 96.65% with the validation dataset and 97.75% with the test dataset. In addition, compared to an expensive model like NASNetLarge, the fused model surpasses its performance, as it only reached an overall accuracy of 77.25% from the validation and 77.62% from the test datasets. Further, as calculated, the model trained with the proposed method only consumed 7.8 GFLOPS, making it relatively cost-efficient compared to its larger counterparts considering its performance in diagnosing the four cases of GI tracts.

## Conclusion

With the growing performance of SOTA DL solutions for GI tract diagnosis, practicality and reproducibility became overlooked. Hence, this method proposed a novel approach to incorporate network compression, layer-wise fusion, residual learning, self-normalization, and regularization to train a model that can compete or perform better than most SOTA solutions without inflating the cost of production. According to the identified findings, a fused model can attain better performance when trained with an MResBlock, SeLU activation, and alpha DO than a model without or that only had the usual DO. Though the proposed method radiated new findings, it also had some drawbacks. Based on the selection and compression method, both did not have a definite approach to identifying a specific model and cut-point layer due to the vastness and complexities of the relationship between the data and the model, prompting that the results may still not be optimal right now. Though these circumstances limited the capability of the proposed method to reach or even determine its optimum state, the straightforwardness and simplicity of the proposed method still yielded a more practical solution than most SOTA and other recent models for automating the diagnosis of GI tracts.

In conclusion, the proposed method opens a promising approach to producing lightweight solutions, specifically for developing a vision-based DL model for GI tract diagnosis. For future studies, others can look into its effectiveness in various medical images to test how it can progress the field to a greater degree and incorporate the concept into other real-time imaging tasks like segmentation and detection.

## Declaration of Competing Interest

The author declares no competing interests. The author declares no known competing financial interests or personal relationships that could have appeared to influence the work reported in this paper.
